# Social immunity in honeybees—Density dependence, diet, and body mass trade‐offs

**DOI:** 10.1002/ece3.4011

**Published:** 2018-04-19

**Authors:** Ben Jones, Emily Shipley, Kathryn E. Arnold

**Affiliations:** ^1^ Fera Science Ltd The National Agri‐Food Innovation Campus York UK; ^2^ Environment Department University of York York UK; ^3^ Present address: Applied Insect Science Ltd Ambrose House, Tanfield Lane Ripon, North Yorkshire UK

**Keywords:** *Apis mellifera*, glucose oxidase, honey bees, insect immunity, prophylaxis

## Abstract

Group living is favorable to pathogen spread due to the increased risk of disease transmission among individuals. Similar to individual immune defenses, social immunity, that is antiparasite defenses mounted for the benefit of individuals other than the actor, is predicted to be altered in social groups. The eusocial honey bee (*Apis mellifera*) secretes glucose oxidase (GOX), an antiseptic enzyme, throughout its colony, thereby providing immune protection to other individuals in the hive. We conducted a laboratory experiment to investigate the effects of group density on social immunity, specifically GOX activity, body mass and feeding behavior in caged honey bees. Individual honeybees caged in a low group density displayed increased GOX activity relative to those kept at a high group density. In addition, we provided evidence for a trade‐off between GOX activity and body mass: Individuals caged in the low group density had a lower body mass, despite consuming more food overall. Our results provide the first experimental evidence that group density affects a social immune response in a eusocial insect. Moreover, we showed that the previously reported trade‐off between immunity and body mass extends to social immunity. GOX production appears to be costly for individuals, and potentially the colony, given that low body mass is correlated with small foraging ranges in bees. At high group densities, individuals can invest less in social immunity than at low densities, while presumably gaining shared protection from infection. Thus, there is evidence that trade‐offs at the individual level (GOX vs. body mass) can affect colony‐level fitness.

## 1 INTRODUCTION

Group living is favorable to pathogen spread due to the increased risk of disease transmission among individuals (Schmid‐Hempel, 1995). In response, animals can alter their immune responses when crowded, a common phenomenon in insects (Wilson & Cotter, 2008). Insects can upregulate their immune system and increase disease resistance at an individual level when in high group densities (density‐dependent prophylaxis), (Bascuñán‐García, Lara, & Córdoba‐Aguilar, 2010; Cotter, Simpson, Raubenheimer, & Wilson, 2010; Krams et al., 2015; Rantala & Roff, 2005; Van Ooik, Rantala, & Saloniemi, 2007). In insects, these costs of mounting immune defenses are demonstrable through trade‐offs with other fitness traits, such as body mass (Bascuñán‐García et al., 2010; Cotter et al., 2010; Krams et al., 2015; Rantala & Roff, 2005; Van Ooik et al., 2007). Eusocial insects, such as ants and bees, exhibit extreme sociality, living in large colonies of hundreds to many thousands of individuals. High density, group living among eusocial insects promotes disease resistance strategies (Hughes, Eilenberg, & Boomsma, 2002; Rosengaus, Maxmen, Coates, & Traniello, 1998; Traniello, Rosengaus, & Savoie, 2002; Ugelvig & Cremer, 2007) and can impact the level of an individual's immunocompetence. For example, eusocial species exhibit greater cuticular antimicrobial peptide (AMP) activity than less social species (Hoggard, Wilson, Beattie, & Stow, 2011; Stow et al., 2007; Turnbull et al., 2011; but see López‐Uribe, Sconiers, Frank, Dunn, & Tarpy, 2016). Furthermore, components of individual immunity show plasticity in response to grouping in some eusocial species, as experimentally grouped bumble bees exhibit different levels of the immune enzyme phenoloxidase (PO) and AMP activity relative to individuals kept in solitude (Richter, Helbing, Erler, & Lattorff, 2012; Ruiz‐González, Moret, & Brown, 2009; but see Armitage & Boomsma, 2010).

An alternative response is that, when in high group densities, insects decrease or at least do not increase, individual immunity but instead benefit from social immune processes that limit pathogen spread and to avoid the costs of the activating the metabolically expensive immune system (Ardia et al. 2012; López‐Uribe et al., 2016; Meunier, 2015). Social immunity, that is antiparasite defenses mounted for the benefit of individuals other than the actor, is predicted to be altered in social groups, but this remains poorly studied. The social immune processes deployed by eusocial insects include removal of infected nest mates (Arathi, Ho, & Spivak, 2006), self‐removal when infected (Alaux, Crauser, Pioz, Saulnier, & Le Conte, 2014; Bos, Lefevre, Jensen, & D'ettorre, 2012; Heinze & Walter, 2010; Rueppell, Hayworth, & Ross, 2010; Ugelvig & Cremer, 2007), depositing corpses outside of foraging ranges (Diez, Deneubourg, & Detrain, 2012), collection of antiseptic saps (Silici & Kutluca, 2005), behavioral fever, where adults sufficiently raise the temperature of the colony to combat brood infections (Starks, Blackie, & Seeley, 2000), and antiseptic enzymatic secretions (Alaux, Ducloz, Crauser, & Le Conte, 2010; Bucekova et al., 2014; White, Subers, & Schepartz, 1963). Honey bees (*Apis mellifera*) secret the antiseptic enzyme and glucose oxidase (GOX) throughout their colonies’ brood food and honey reserves, thereby providing social immunity to nest mates. Briefly, GOX is produced in the hypopharyngeal glands and catalyzes the oxidation of β‐d‐glucose to gluconic acid and hydrogen peroxide (H_2_O_2_) (Alaux et al., 2010; Bucekova et al., 2014; Sano et al., 2004; Santos et al., 2005; White et al., 1963). H_2_O_2_ acts an antiseptic inhibiting pathogen growth in larval food of honeybees (e.g., Brudzynski, 2006) with a recent study showing that GOX is produced constitutively rather than being induced as a response to pathogen pressure (López‐Uribe, Fitzgerald, & Simone‐Finstrom, 2017).

No studies have directly investigated the effect of group density on a physiological social immune response in a eusocial insect. It remains unknown as to whether a physiological social immune response would increase, in order to combat heightened pathogen pressure (Bascuñán‐García et al., 2010; Cotter et al., 2010; Krams et al., 2015; Rantala & Roff, 2005; Van Ooik et al., 2007), or decrease in individuals living in high group densities, as the benefits are of social immunity are shared (López‐Uribe et al., 2016; Meunier, 2015). For example, as more individuals become available to contribute to some constitutive level, less GOX production might be needed per capita (Duarte et al., 2016). The primary aim of this study was to therefore investigate whether the previously reported density‐dependent plasticity of individual immunity extends to a social immune response in a eusocial insect. Specifically, we test whether honey bees caged in high and low group densities exhibit different levels of GOX activity. Our second aim was to investigate the previously reported trade‐off between immunocompetence and body mass. This trade‐off could have colony, as well as individual, level consequences as in honeybees; body mass is correlated with foraging range (Greenleaf, Williams, Winfree, & Kremen, 2007), an important fitness trait for the colony. Finally, we also tested the effects of group density on feeding behavior (realized diet) as diet is known to affect immunocompetence in honeybees (Alaux et al., 2010).

## 2 METHODS

### 2.1 Honey bee provenance and husbandry

Frames containing capped worker brood were obtained from four colonies of *A. mellifera*, located at the National Bee Unit apiary, York, UK, and incubated at 33–34°C in constant darkness to mimic colony conditions. After 24 hr, newly emerged honeybees were collected and caged in plastic containers (11.4 cm diameter × 7.7 cm) in groups of either sixty (high density, *n* = 16 cages) or six bees (low density, *n* = 16 cages). Due to availability of emerging bees, for each group density, two colonies each provided three cages of bees and the remaining two colonies each provided five cages of bees. All bees had access to syrup (50% w/v sucrose/distilled H_2_O) and pollen via feeders (modified 1.5 ml microcentrifuge tubes) inserted into the cages (Na'vi Organics Ltd. © mixed pollen, mixed to a paste of 50% v/w distilled H_2_O). Syrup was accessible via a small hole cut into the feeder. Pollen was accessible via a large cut “trough” in the side of the feeder. In order to combat any effect of competition for the syrup feeder, two syrup feeders were inserted into all cages. The food was stored at 7°C prior to use and until the end of the experiment. Cages were incubated under constant conditions as above and randomly positioned every 24 hr to account for a position effect.

To mitigate any concentrating effect from evaporation, feeders were replaced every 24 hr for 10 days, and daily consumption was calculated by subtracting the postweight of the feeders from the preweight. Daily evaporation was measured from feeders in three cages devoid of bees and subtracted from the daily consumption. Where the evaporation rate was greater than consumption, the values were zeroed, and any data from day or cages where spillages occurred were omitted from analyses.

### 2.2 Assaying social immunity

Analysis of GOX activity was determined photospectromically using a Tecan Infinite^®^ 200 PRO plate reader and the Amplex^®^ Red Glucose/Glucose Oxidase Assay Kit (MP 22189) (Molecular Probes^™^, 2006). At the end of the 10‐day experiment, the bees were killed and weighed, and individual bee heads were ground in 100 μl PBS solution. The individual samples were centrifuged at 4°C for 10 min at 6037.2g, and a 50 μl of the supernatant was collected and pooled with the other samples from that cage. Pooled cage samples were added to a 96‐well plate. Each well contained a 20 μl aliquot of the pooled cage sample and 30 μl of reaction mixture consisting of Amplex^®^ Red reagent, horseradish peroxidase and glucose. Each sample was replicated twice, and both positive and negative GOX controls were included on the plate (Positive: 20 μl of 0.39 mU/μl + 30 μl reaction mixture; Negative: 20 μl PBS + 30 μl reaction mixture). The reactions were incubated at room temperature for 30 min, with the absorbance measured at 560 nm every 40 s and a shaking duration of 1‐s between reads. The maximum linear slope of the reaction over 13 min (the *V*_max_ value) was used as a level of GOX activity. The mean negative control *V*_max_ value was subtracted from the each of the mean sample replicate *V*_max_ values.

### 2.3 Body mass

In order to measure the individual cost of GOX activity, the mean frozen body weight of three randomly selected bees from each cage, prior to decapitation for GOX analysis, was used as a proxy for body mass (Laughton et al. 2011).

### 2.4 Statistical analyses

All statistical analyses were performed in R 3.0.2 (R Core Team, 2017). The effects of group density, colony, and their interaction on the activity of GOX, total consumption, and body weight were analyzed with ANOVA's. Repeated daily consumption was analyzed with a mixed effects model using the lme4 package with cage and treated as the random effect Pinheiro et al. (2017). Models were fitted up to all two‐way interactions, and significance was determined by assessment of the minimal adequate model. Day was treated as a categorical variable to allow post hoc comparisons of consumption on different days by bees in the high and low group densities with Tukey corrections within the “lsmeans” package (Lenth, 2016). All assumptions of normality and heterogeneity were checked by visual inspection of the residuals.

## 3 RESULTS

### 3.1 Glucose oxidase activity and body mass

**Figure 1:**
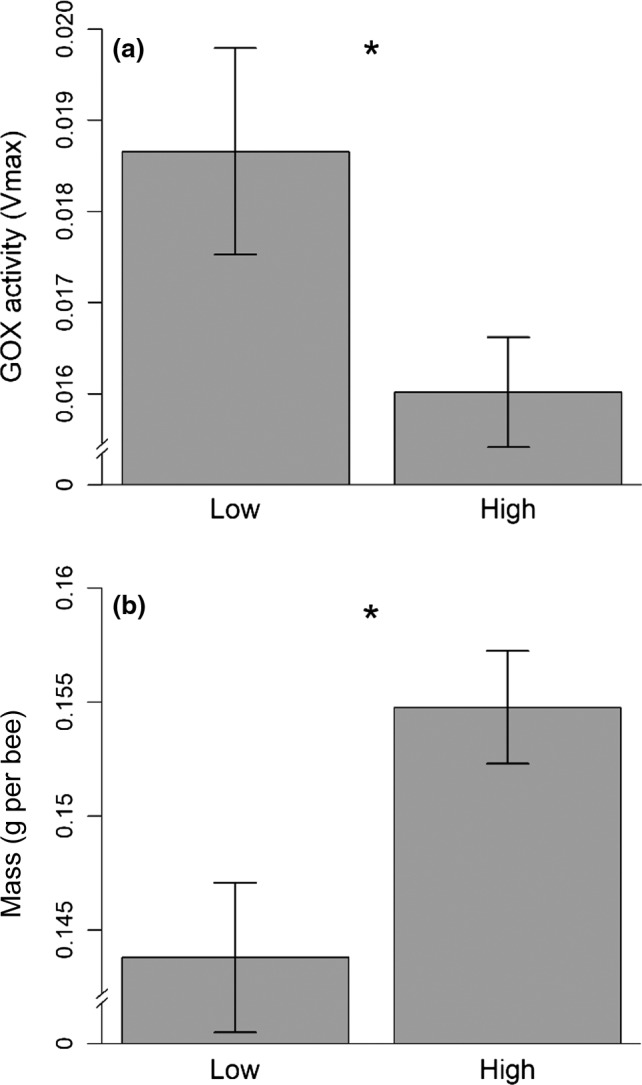
Glucose oxidase (GOX) activity per bee (a) and the frozen body mass (g per bee), (b) for honeybees caged in high (60 bees, *n* = 16 cages) and low (6 bees, *n* = 16 cages) group densities. Error bars denote *SEM*, and stars show significant differences (*p* < .05)

Group density affected the activity of GOX, which was higher in bees kept in the low group density compared to the high group density (ANOVA: *F*_1,27_ = 5.09, *p = *.03, Figure 1a). The activity of GOX also varied among colonies (ANOVA: *F*_3,27_ = 3.04, *p = *.045) but there was no significant interaction between colony and group density (ANOVA: *F*_3,24_ = 0.93, *p = *.44) on activity of GOX. In terms of body mass, bees in the high group density were 7% heavier (mass = 0.155 g ± 0.002 *SE*) than bees in the low group density (mass = 0.144 g ± 0.003 *SE*; ANOVA: *F*_1,30_ = 7.13, *p = *.01, Figure 1b). Body mass did not vary among the colonies (ANOVA: *F*_3,27_ = 1.1, *p = *.36) and there was no significant interaction between colony and group density (ANOVA: *F*_3,24_ = 1.9, *p = *.15).

### 3.2 Consumption

**Figure 2:**
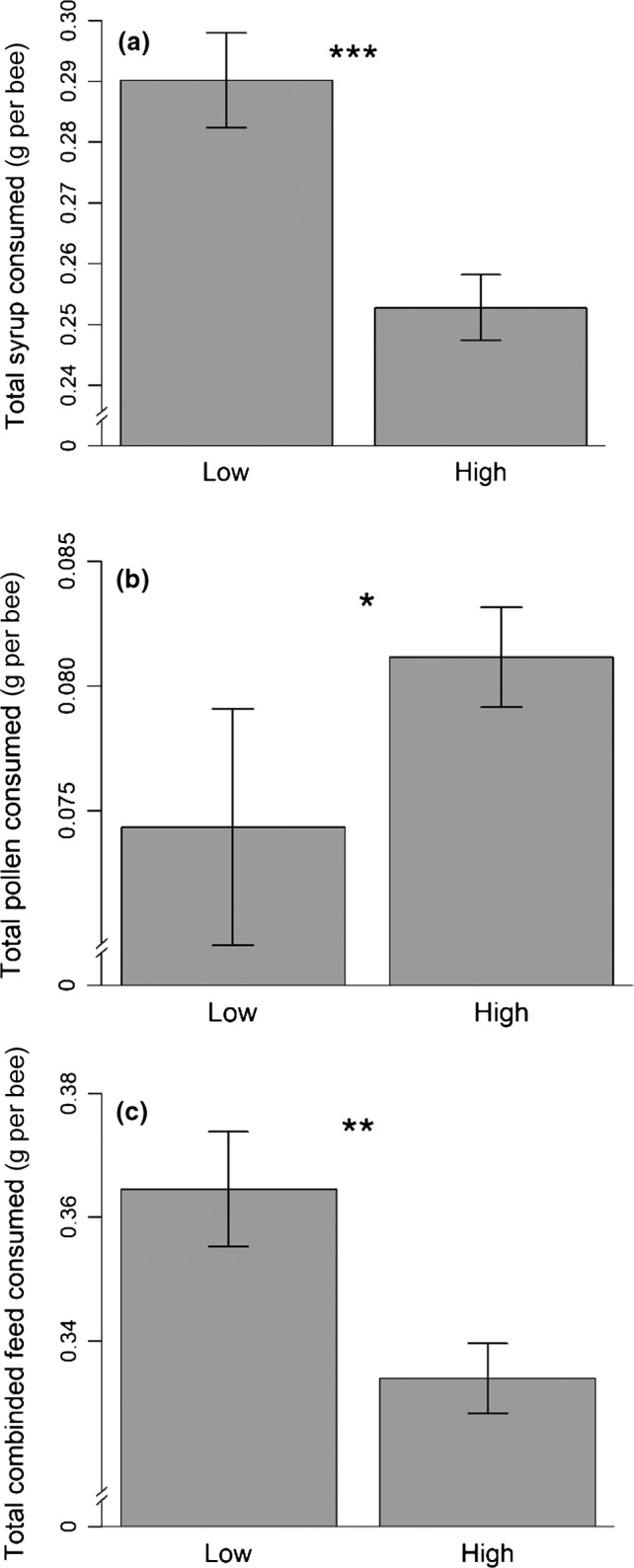
Total consumption of syrup (a), pollen (b), and combined feed (c) (g per bee) by honeybees caged in high (60 bees, *n* = 15 cages) and low (6 bees, *n* = 8 cages) group densities, after 10 days. Cages, where spillages occurred, were not included. Error bars denote *SEM*, and stars show significant differences (*p < .05, **p < .01, ***p < .001)

The total consumption of pollen per bee over 10 days was lower by bees in the low group density, whereas the inverse was true of syrup consumption, leading to more total combined feed was consumed by bees in the low‐density group (ANOVAs: Syrup, *F*_1,17_ = 21.46, *p < *.001, Figure 2a; Pollen, *F*_1,17_ = 5.18, *p = *.04, Figure 2b; Combined total, *F*_1,17_ = 15.89, *p < *.001, Figure 2c). The total consumption of pollen and the total combined feed also varied among the source colonies (ANOVAs: Pollen, *F*_3,17_ = 4.10, *p = *.02; Combined total, *F*_3,17_ = 7.2, *p = *.003). Consumption of syrup was not affected by the colony (*F*_3,17_ = 3.09 *p = *.06), and there were no significant interactions between colony and group density on consumption (Syrup: *F*_3,14_ = 0.36, *p = *.78, Pollen: *F*_3,14_ = 2.6, *p = *.09, Combined total: *F*_3,14_ = 0.99, *p = *.43).

Daily consumption of both syrup and pollen varied significantly over time. Syrup consumption generally increased, while pollen consumption initially increased, but then decreased over 10 days. However, these time trends were dependent on the group density for consumption of both syrup (Day × Group density, χ^2^ (9) = 48.63, *p* < .001) and pollen (Day × Group density, χ^2^ (9) = 34.36, *p* < .001, see Figure S1a,b). Bees consumed more pollen on the first 2 days in the high group density, compared to bees in the low group density (high vs. low: Day 1, *p* = .002, Day 2, *p* < .001). Consumption of syrup was higher in the low group density on day two (*p* < .001), reduced on day four (*p* = .004) and then higher over the last 3 days, compared to bees in the high group density (Day 8, *p* = .002, Day 9, *p* = .02, Day 10, *p* < .001). The interaction between the colony and time also affected daily consumption of syrup (χ^2^ (27) = 40.53, *p* = .046) and pollen (χ^2^ (27) = 43.58, *p* = .02). However, post hoc testing revealed no clear patterns between colonies on different days (see Table S1). Daily consumption was not affected by the interaction between the group density and colony for either syrup (χ^2^ (3) = 1.95, *p* = .58) or pollen (χ^2^ (3) = 3.36, *p* = .34).

## 4 DISCUSSION

Our results provide the first experimental evidence that group density affects a social physiological immune response in a eusocial insect. Furthermore, we found evidence for a group density mediated trade‐off between GOX activity and body mass, supporting previous findings that immunity and body mass are under resource allocation and traded‐off in insects (Bascuñán‐García et al., 2010; Cotter et al., 2010; Krams et al., 2015; Rantala & Roff, 2005; Van Ooik et al., 2007). The activity of GOX increased in bees caged in a low group density, compared to bees caged in a high group density, despite bees in the high group density having increased mass relative to bees in the low group density.

We suggest that our findings represent a compensatory response by honey bees to low group densities, whereby more GOX is produced per capita as fewer individuals become available for GOX production and vice versa, producing a homeostatic effect at the colony level. A recent study by Duarte et al. (2016) reported a similar phenomenon in the burying beetle, *Nicrophorus vespilloides*, where mothers reduced their contribution of social immunity (AMP exudates, smeared on the carcass that they rear brood on) when raising large broods, irrespective of parental care. As the larvae themselves contribute to AMP exudates, the reduction was potentially explained by mothers adjusting to contributions made by larvae (Duarte et al., 2016). Thus, there is evidence to suggest that social immunity defenses in invertebrates are produced to some functional level by individuals, with benefits shared across group members.

Alternatively, decreased GOX production in high group densities may represent a trade‐off with components of individual immunity, such as PO and AMP's. The effects of group density on the individual immune responses in honeybees are unknown. However, three studies have directly investigated the effects of sociality on individual immunity in other Hymenoptera with conflicting results. Group density had no effect on the PO response in ants (Armitage & Boomsma, 2010). In contrast, two studies of individual immunity in *Bombus terrestris* demonstrated that levels of PO and AMP's were altered at both the phenotypic and gene expression levels in bumble bees kept in groups, compared to individuals kept in solitude. Group size increased phenotypic expression of PO, but decreased AMP activity, whereas the opposite trend was found for gene expression levels (Richter et al., 2012; Ruiz‐González et al., 2009). Although results were opposing, the differences were attributed to potential variations in methodology between the two studies or unknown factors between the expression of the selected genes and the downstream physiological response (Richter et al., 2012). Differences between species and studies could also be attributed to varying experimental timescales. The experiments by Ruiz‐González et al. (2009) and Richter et al. (2012) were run for 8 and 7 days, whereas in the experiment by Armitage and Boomsma (2010), PO was measured after 48 hr, which may have not provided sufficient time to detect any effect of group density (Armitage & Boomsma, 2010). Our observed decrease in GOX production in high group densities may therefore be accompanied by an upregulation of components of individual immunity, not investigated here, supporting the density‐dependent prophylaxis hypothesis.

Reduced growth in individuals displaying increased social immune activity might not be surprising, given the direct metabolic cost of immune activation (Ardia et al. 2012). However, evidence exists for an underpinning dietary mechanism behind our observed trade‐off. Cotter et al. (2010) demonstrated that trade‐offs between multiple fitness traits in the moth *Spodoptera littoralis*, including larval mass and components of individual immunity, can be mediated by diet, in that no single diet could optimize all fitness traits. In our study, consumption behavior followed the expected, age‐related, pattern. Honeybees first increased pollen consumption as after emergence pollen is required for glandular tissue development. This time represents the onset of nursing duties when high protein larval food is produced by the hypopharyngeal glands. As honeybees age, they decrease pollen consumption, while increasing carbohydrate consumption, in line with the transition from the domestic nursing phase to foraging duties (Ament, Wang, & Robinson, 2010; Crailsheim & Stolberg 1989, DeGrandi‐Hoffman et al. 2010, Di Pasquale et al., 2013; Haydak, 1970; Paoli et al., 2014; Pernal & Currie, 2000; Sagili, Pankiw, & Zhu‐Salzman, 2005; Toth, Kantarovich, Meisel, & Robinson, 2005).

The impact of diet on insect immunity has been the focus of numerous studies (Povey, Cotter, Simpson, & Wilson, 2014, for review, see Ponton et al., 2013), including honeybees (Alaux, Dantec, Parrinello, & Le Conte, 2011; Alaux et al., 2010; Szymaś & Jędruszuk, 2003). Alaux et al. (2010) found that 10‐day‐old honeybees had increased GOX activity when they had access to dietary pollen. In our study, all bees had access to pollen. Thus, a potential explanation is that the relationship between GOX and consumption is not entirely reliant on the availability of pollen and GOX production might also be driven by carbohydrate consumption. If our observed dietary shift affected GOX activity, a potential explanation is that honeybees selected a carbohydrate‐biased diet to promote GOX activity when needed in low group densities. Alternatively, any dietary‐mediated effect on GOX may be less direct. For example, group density affects juvenile hormone (JH) in honeybees, which regulates temporal polyethism. Foragers contain more JH than nurse bees, and JH is inversely related to group size (Huang & Robinson, 1992). It is therefore possible that the bees caged in the low group density precociously adopted a forager‐like, carbohydrate‐biased diet.

We do not think that the decreased total uptake from the syrup feeder by bees caged in the high group density was an artifact resulting from competition, as the opposite trend was seen for pollen consumption and more syrup was not consistently consumed in the low group density. Indeed, bees consumed *more* syrup in the high group density on day four and similar amounts for five of the experimental days (Figure S1a). Further work is clearly needed to explain our observed effect of group density on feeding behavior. A carbohydrate‐biased diet could also explain our observed difference in mass between the two group densities as, although bees in the high group density consumed less food in total, they also consumed the most pollen. It is perhaps not surprising that these bees also had increased mass, given that pollen is required for so much of the honeybee's physiological development, including the hypopharyngeal glands (Crailsheim et al., 1992; DeGrandi‐Hoffman et al. 2010, Di Pasquale et al., 2013; Pernal & Currie, 2000), internal organs (Hagedorn & Moller 1968; Haydak, 1970; Hoover, Higo, & Winston, 2006; Pernal & Currie, 2000), and fat bodies (Alaux et al., 2010; Haydak, 1970), as well as increased dry body weight, nitrogen content (De Groot, 1953), overall body mass (Crailsheim, 1990; Hoover et al., 2006) and hemolymph protein levels (Cremonz, De Jong, & Bitondi, 1998).

## 5 CONCLUSIONS

Decreasing the group density of caged honeybees increased the activity of the social immune enzyme, GOX per capita, and decreased body mass, suggesting a compensatory response and providing evidence for a trade‐off between these two traits. Our findings might be driven by an underlying dietary mechanism as bees fed differentially in the two group densities; bees caged in a low group density consumed a carbohydrate‐biased diet and consumed more in total. Future work should investigate (1) whether group density affects other parameters of immunity in eusocial insects, such as the PO system, AMP's, and potential trade‐offs with body mass and between social and individual immune responses and (2) the effects of group density on immunocompetence under controlled dietary regimes. Our study provides evidence for the multifaceted costs associated with small colony size for honeybees (Budge et al., 2015), at both the individual and colony level.

## CONFLICT OF INTEREST

None declared.

## AUTHOR CONTRIBUTIONS

Ben Jones conceived the ideas and designed methodology; Emily Shipley collected the data; Emily Shipley and Ben Jones analyzed the data; Kathryn Arnold and Ben Jones led the writing of the manuscript.

## DATA ACCESSIBILITY

Data available from the Dryad Digital Repository (Jones et al. 2018).
