# Supplementary material for: Social immunity in honeybees—Density dependence, diet, and body mass trade‐offs
Source: Ecol Evol. 2018 Apr 19;8(10):4852–9. doi: 10.1002/ece3.4011 (PMC5980322; doi:10.1002/ece3.4011)
Supplement: Supplementary file 1 [file ECE3-8-4852-s001.docx]

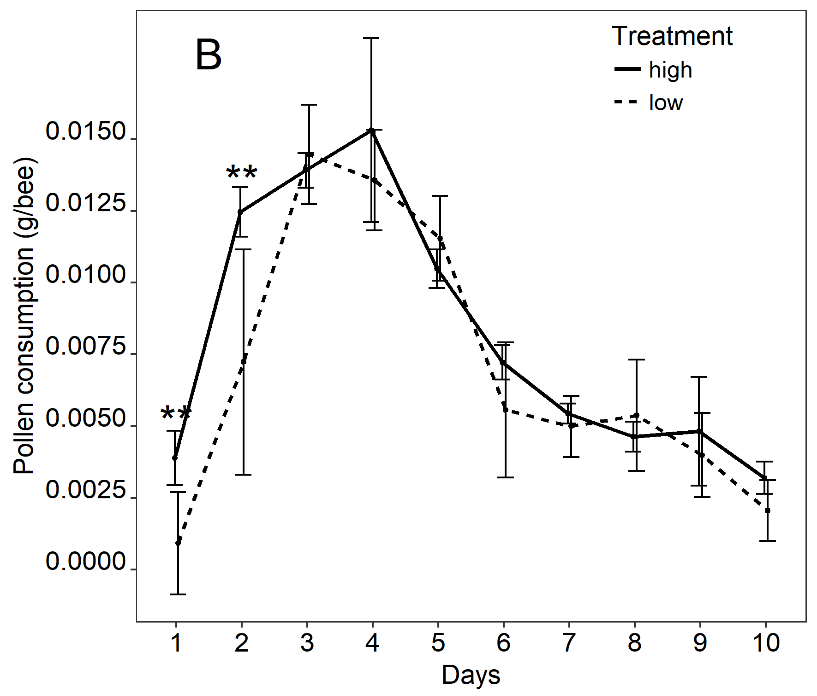
*
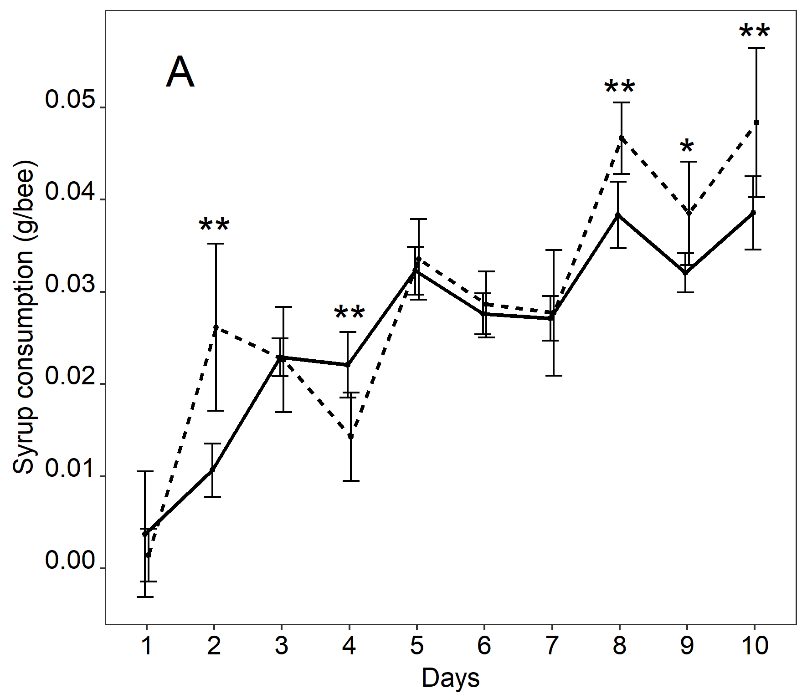
Supporting Information*

*Figure S1. Syrup (50% sucrose/dH_2_O), (A) and pollen (B) consumption per bee (g bee^-1^) by honey bees caged in High (60 bees, n=16 cages) and Low (6 bees, n=12-16 cages) group densities over ten days. Data were not included on days where food spillages occurred in a cage. Error bars show 95% C.I. and stars show significance differences between group densities on each day (* p<0.05, ** p<0.01).*

*Table S1. Significant pairwise colony comparisons of consumption of pollen and syrup on different days*.

| **Feed** | **Day** | **Pairwise comparison** | ***p*** |
| --- | --- | --- | --- |
| **Pollen** | Day 2 | Colony 1 < Colony 4 | 0.005 |
|  |  | Colony 1 < Colony 3 | 0.001 |
|  | Day 4 | Colony 1 > Colony 2 | 0.01 |
|  |  | Colony 2 < Colony 4 | <0.001 |
|  | Day 5 | Colony 2 < Colony 4 | 0.02 |
|  | Day 6 | Colony 4 > Colony 2 | 0.04 |
| **Syrup  (50% w/v sucrose sol)** | Day 10 | Colony 1 > Colony 2 | <0.001 |
|  |  | Colony 1 > Colony 4 | 0.02 |
|  |  | Colony 2 < Colony 3 | 0.02 |

All other pairwise colony comparisons on all days were non-significant (p>0.05). *p* values were obtained with Tukey corrections within lsmeans package (Lenth 2016).
